# Piezoelectric MEMS microphones based on rib structures and single crystal PZT thin film

**DOI:** 10.1038/s41378-024-00767-5

**Published:** 2024-11-08

**Authors:** Zhiwei You, Jinghan Gan, Chong Yang, Renati Tuerhong, Lei Zhao, Yipeng Lu

**Affiliations:** School of Integrated Circuits, Peking University, Beijing, China

**Keywords:** Electrical and electronic engineering, Sensors

## Abstract

In this study, a controllable mass‒frequency tuning method is presented using the etching of rib structures on a single-crystal PZT membrane. The rib structures were optimized to reduce the membrane mass while maintaining the stiffness; therefore, the center frequency could be increased to improve the low-frequency bandwidth of microphones. Additionally, this methodology could reduce the modulus and improve the sensitivity for the same resonant frequency, which typically indicates the maximum acoustic overload point (AOP). The PZT film was chosen because of its greater density; the simulation results showed that PZT could provide a greater frequency tuning (24.9%) compared to that of the AlN film (5.8%), and its large dielectric constant enabled the optimal design to have small electrodes at the maximum stress location while mitigating the sacrificial capacitance effect on electrical gain. An analytical model of rib-structure microphones was established and greatly reduced the computing time. The experimental results of the impedance tests revealed that the center frequencies of the six microphones shifted from 74.6 kHz to 106.3 kHz with rib-structure inner radii ranging from 0 μm to 340 μm; this result was in good agreement with the those of the analytical analysis and finite element modeling. While the center frequency greatly varied, the measured sensitivities at 1 kHz only varied within a small range from 22.3 mV/Pa to 25.7 mV/Pa; thus, the membrane stiffness minimally changed. Moreover, a single-crystal PZT film with a (100) crystal orientation and 0.24-degree full width at half maximum (FWHM) was used to enable differential sensing and a low possibility of undesirable polarization. Paired with a two-stage differential charge amplifier, a differential sensing microphone was experimentally demonstrated to improve the sensitivity from 25.7 mV/Pa to 36.1 mV/Pa and reduce the noise from −68.2 dBV to −82.8 dBV.

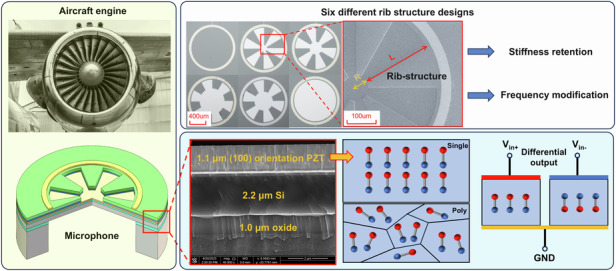

## Introduction

Microphones based on MEMS technology have experienced great interest across various fields, such as consumer electronics and medical applications. Traditional applications such as mobile phones and hearing aids have relied on film deformation to convert acoustic signals into electrical signals. The prevailing design focuses on increasing sensitivity and reducing noise within the audio frequency band to increase the signal-to-noise ratio (SNR)^1–5^. MEMS microphones have also demonstrated substantial promise in high sound pressure level (SPL) applications such as aircraft noise assessment and engine condition monitoring^6–8^. However, microphones deployed in high-SPL environments often encounter significant harmonic distortion due to nonlinear behavior^9^. The maximum acceptable distortion is generally identified as 10%^10^, and the corresponding SPL is described by the AOP, which evaluates microphone performance under high SPL. Unfortunately, designers often face a trade-off between sensitivity and AOP, as these metrics have opposing stiffness requirements, resulting in significant bandwidth variations. For example, microphones for aeroacoustic applications are typically operated within a bandwidth of 20 Hz–70 kHz, whereas those for audio applications are generally operated from 20 Hz to 20 kHz^6,7^. In this case, investigating frequency and bandwidth modification based on the stiffness and mass variation can provided significant insights for microphone design^11^.

Traditional microphones mainly consist of moving coil and electret types and are strongly constrained by physical dimensions. They inherently suffer from shortcomings such as high-frequency output attenuation, substantial background noise, low integration density, and limited applicability in consumer electronics and biomedicine^12–14^. MEMS microphones have emerged as a better option owing to their cost-effectiveness in mass production^15^. MEMS microphones can be categorized into capacitive, piezoresistive, and piezoelectric types on the basis of different mechanisms. Compared with capacitive and piezoresistive transduction principles, the self-generating charge mechanism of piezoelectric materials provides energy efficiency advantages^16,17^. Moreover, the simplistic structure of the single-layer membrane ensures better linearity and minimal harmonic distortion at high SPL. Furthermore, biomimetic piezoelectric microphones based on the fruit fly eardrum can be used to create directional microphones that can achieve directional sound reception on a single device rather than a microphone array^18–20^.

Since the inception of the first piezoelectric MEMS microphone, numerous options for piezoelectric films have been investigated^21^, and AlN, ZnO, and PZT are the most commonly used. Although AlN and ZnO have better receiving sensitivities, owing to their small dielectric constants, they usually need large electrode areas to obtain reasonably large characteristic capacitances to mitigate the performance reduction caused by the parasitic capacitance and therefore lose the flexibility of the optimal electrode design. PZT has a large piezoelectric coefficient and dielectric constant^10^, which allows a small area of the electrode to be distributed in the stress concentration area or form a series connection to improve the sensitivity^22–24^. Moreover, the ferroelectric polarization mechanism of PZT facilitates differential sensing output. By constructing two reverse-polarized PZT films of identical structure, a differential output can be achieved on a single device, enabling common-mode noise suppression and SNR enhancement. The PZT also has a relatively high density, resulting in more significant frequency modifications via rib structures. Additionally, the performance of a PZT thin film heavily relies on its deposition method to obtain an aligned crystal orientation and high piezoelectric coefficients.

In this work, we present a series of piezoelectric MEMS microphones fabricated from high-quality single-crystal PZT thin films. By incorporating rib structures into piezoelectric films, we demonstrated center frequency tuning by reducing mass and maintaining the stiffness and sensitivity. Furthermore, we constructed a finite-degree-of-freedom analytical model with high computational efficiency based on circular thin films with rib structures to guide sensor design and frequency tuning; this model was verified by the experimental results. Additionally, leveraging the polarization characteristics of single-crystal PZT, we utilized oppositely polarized PZT films and achieved differential sensing with improved sensitivity and mitigated noise levels. In this study, a frequency modification method with minimal changes to the stiffness is developed by constructing rib structures on the membrane, and the potential of using single-crystal PZT to increase microphone sensitivities with differential outputs is shown.

## Device design and fabrication

### Working principles

**Fig. 1:**
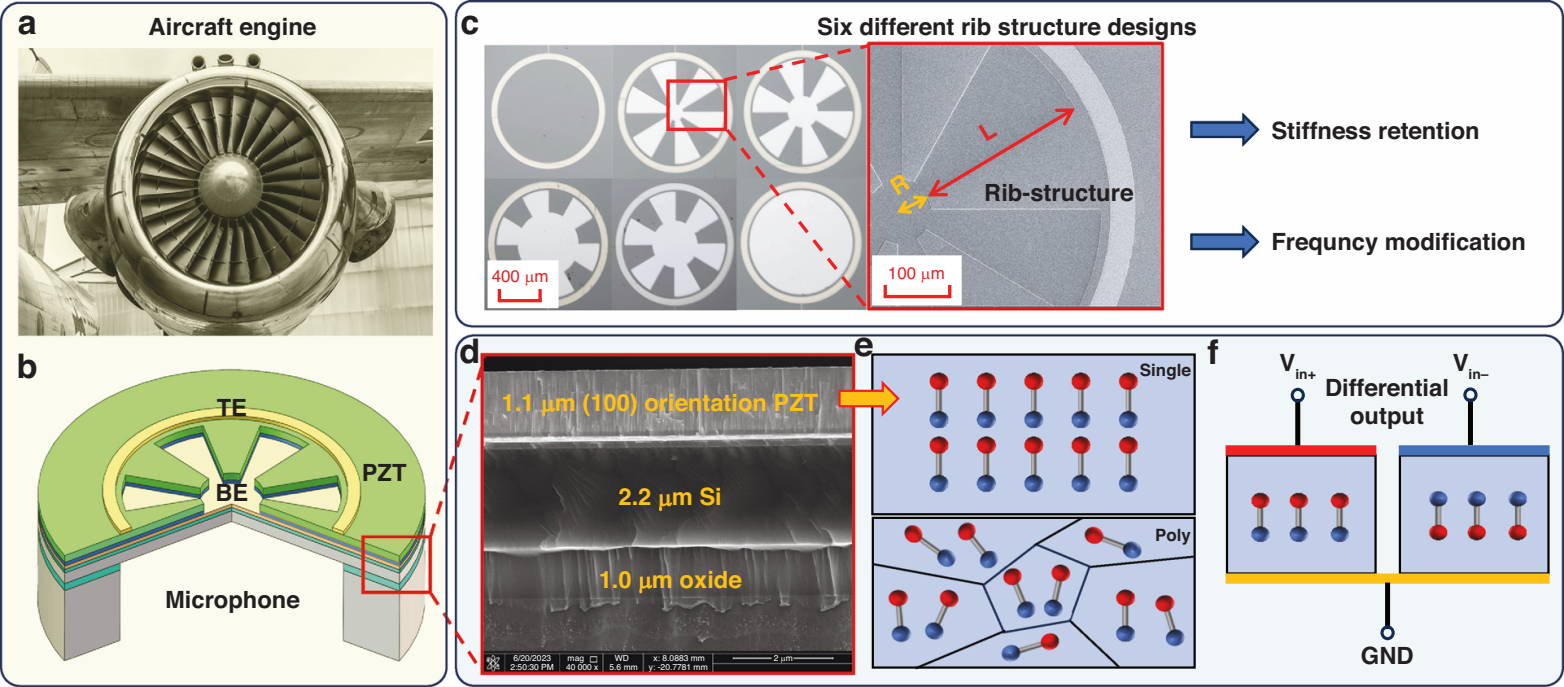
Schematic of the rib-structured microphone. **a** High SPL application for microphones such as aircraft engine. **b** Diagram of the proposed microphone. **c** Optical and SEM images of the six different rib-structure designs. **d** SEM image of the membrane multilayers, showing a 1.1 μm PZT layer with an orientation of (100). **e** The model of the single crystal PZT and polycrystal PZT after poling. **f** The differential output based on the reverse polarization of single crystal PZT

MEMS piezoelectric microphones operate based on the principle of the piezoelectric effect, wherein a thin film comprising piezoelectric layers and passive layers undergoes deformation under sound pressure and induces stress. This stress generates electrical charges, which are collected by upper and lower electrodes connected with a charge amplifier^25^. The electrodes are strategically positioned at the highest stress concentration location, typically along the edges of the circular film. As illustrated in Fig. 1a, one potential application scenario is aeroacoustic application, such as engine monitoring or fuselage noise measurement. These applications usually require microphones to maintain good linearity under a high SPL of up to 170 dB, which cannot be supported by most microphones because of severe distortion. A diagram of the designed microphone is shown in Fig. 1b.

To attain frequency modification while minimizing the variation in membrane stiffness, portions of the PZT layer are etched to reduce mass, resulting in a distinctive rib structure, as shown in Fig. 1c. In this configuration, approximately 15% of the edge length is retained to maintain stiffness. A cavity radius of 400 μm is selected to ensure flat output and high sensitivity within the audio band with a resonant frequency of 76.4 kHz. Furthermore, the electrode is meticulously designed for maximum sensitivity. Six rib structures were designed to investigate frequency and bandwidth modifications through rib structures with varying side lengths and inner radii and validate the analytical model. These structures correspond to inner radii of 0 μm, 50 μm, 100 μm, 150 μm, 200 μm, and 340 μm. Each device incorporates six ribs, and each rib spans an angle of 30 degrees. A 1.1 μm single-crystal PZT layer is used to ensure better polarization and a low risk of depolarization. To further enhance the SNR, a differential output based on the reverse polarization of a single-crystal PZT is shown in Fig. 1d–f.

### Analytical model simulation

To provide clear guidance on the design procedure of our rib-structured device with limited computing resources, we implemented an analytical-based numerical analysis of the microphone design from the perspective of structural mechanics and material properties and verified the feasibility of frequency and bandwidth modification from the finite element modeling (FEM) results. Previous theoretical studies focused on circular thin films with standard peripheral support^26^. However, due to the lack of rotational symmetry, special membrane structures usually introduce additional degrees of freedom and cause challenges in deriving a simple analytical solution of the differential equation of stiffness and resonance frequency. In this study, we convert the problem of solving partial differential equations into a variational problem that can be solved by using the optimization method. Furthermore, the discretizing of the variational problem is shown by using the spline function to fit the real radial curve; this ensures that the search space of the fitting curves is sufficient to obtain sufficiently accurate numerical results for the design procedure and that the number of optimization parameters is limited for quick computation. As a result, a Python program is used, which greatly accelerates the resonant frequency analysis procedure with respect to the commonly used FEM simulation (from ~ 10 min to less than 0.1 s) and reduces the computational resource needs at the same time. In addition, the influence of film stress is added to the equation to provide a more accurate and feasible solution for addressing residual stress concerns. Additionally, the validation of our program is tested in several designed degenerate models where a simple analytical solution exists, and we obtain good agreement between our numerical results and previous theoretical results^26,27^.

For a circular film with completely clamped edges, the characteristic frequency ${w}_{0}$ is determined by the stiffness $D$ and the surface density $\sigma$ of the film, with the following equation^28^:

1 $${D\nabla }^{4}{w}_{0}=\sigma {\omega }^{2}{w}_{0}$$

When a uniform stress T is applied to the film, the equation can be rewritten as follows:

2 $${D\nabla }^{4}{w}_{0}+T{\nabla }^{2}{w}_{0}=\sigma {\omega }^{2}{w}_{0}$$

Considering the boundary conditions at the edge of the film, the resonant frequency is zero because of the fully clamped edges. The frequency is radially continuous, such the deviation of the frequency to the radius is also zero; then, the following equations apply:

3 $${w}_{0}=0$$

4 $$\frac{\partial {w}_{0}}{\partial r}=0$$

For a uniform circular film, the resonant frequency of the film without stress can be expressed as follows:

5 $$\omega =\left(\frac{{\alpha }^{2}}{{r}_{0}^{2}}\right)\sqrt{\frac{D}{\sigma }}$$

where $\omega$ is the resonant frequency, $\alpha$ is the coefficient, ${r}_{0}$ is the radius of the film, $D$ is the stiffness of the film, and $\sigma$ is the surface density of the film. In the case of high internal plane stress^29^, the coefficient of the film is no longer uniform, and the equation is rewritten as follows:

6 $$\omega =\frac{\alpha \beta }{{r}_{0}^{2}}\sqrt{\frac{D}{\sigma }}$$

where $\beta$ is the stress parameter number.

7 $$\beta =\sqrt{{\alpha }^{2}+\frac{T{a}^{2}}{D}}$$

For a uniform circular membrane, both $\alpha$ and $\beta$ satisfy the following equation:

8 $$\frac{\alpha {J}_{1}\left(\alpha \right)}{{J}_{0}\left(\alpha \right)}+\frac{\beta {I}_{1}\left(\beta \right)}{{I}_{0}\left(\beta \right)}=0$$

where ${J}_{1}\left(\alpha \right)$, ${J}_{0}\left(\alpha \right)$, ${I}_{1}\left(\beta \right)$ and ${I}_{0}(\beta )$ are the transcendental cylindrical functions^30^. For a uniform circular film, this equation is adequate for solving α; however, due to the C6-symmetric three-layer structure of a rib-structure film, the calculation complexity greatly increases. On one hand, there is a superposition of angular column functions. On the other hand, there is a continuity equation between the different radial and poloidal interfaces (4 for each interface). Thus, truncation is considered. A 12n-order determinant zero problem needs to be solved to obtain the analytical expression (n is a positive integer), and every element involved in the determinant is a column function (transcendental function); even if the determinant is assembled using this solution, only numerical solutions can be attained.

Therefore, an approximate solution appears to be a more reasonable choice because its analytical version is trivial and complex yet still not sufficiently straightforward unless the numerical result is calculated. Here, the piecewise polynomial function is used to approximate the configuration of the membrane vibration, and the original eigenvalue problem is transformed into an optimization problem. Specifically, the membrane configuration that causes the integral to obtain the minimum value is solved.

9 $$\int D{\left({\nabla }^{2}{w}_{0}\right)}^{2}+T{\left(\nabla {w}_{0}\right)}^{2}{dS}=\int \sigma {\omega }^{2}{w}_{0}^{2}{dS}$$

The space in which we search for the solution is as follows:

10 $${w}_{0}={\sum }_{n}{W}_{n}(r)\sin (n\theta )$$

Here, we set ${W}_{n}\,(r)$ as a cubic Hermite polynomial determined by the values and derivatives of a series of interpolation points and set those values as discrete parameters. The $w$ corresponding to the null solution of the determinant of the optimization numerical problem is both the eigenfrequency, and the determinant calculation is performed using the Python program.

The final result also satisfies the vibrations of the two parameters of $\left\{{D}_{1},{\sigma }_{1}\right\}$ and$\{{D}_{2},{\sigma }_{2}\}$, as follows:

11 $$\omega =\frac{f\left(\frac{{D}_{1}}{{D}_{2}},\frac{{\sigma }_{1}}{{\sigma }_{2}}\right)}{{r}_{0}^{2}}\sqrt{\frac{{D}_{1}}{{\sigma }_{1}}}$$

The numerical results for function $f({D}_{1}/{D}_{2},{\sigma }_{1}/{\sigma }_{2})$ are provided by the Python program. For the reasons mentioned above, we believe that our analytical-model-based numerical program can provide adequate guidance for the design of microphones and PMUTs with adequate accuracy and efficiency.

**Fig. 2:**
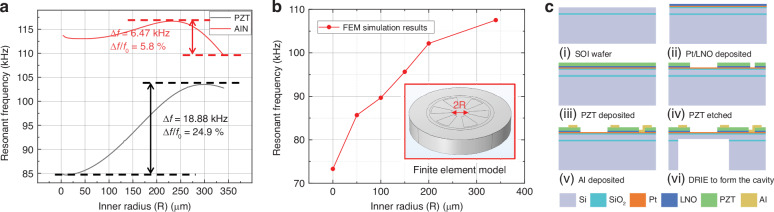
Analytical and FEM simulation results together with the process flow. **a** Analytical modeling results of resonant frequency vs. rib-structure inner radius (4 μm to 340 μm) of PZT and AlN. **b** FEM results of resonant frequency vs. rib-structure inner radius. **c** The schematic diagram of the process flow of proposed microphones

According to the analytical model mentioned above, the modification effect of the rib structure on the frequency is significantly influenced by the stiffness and density of the piezoelectric material. To ascertain a more pronounced frequency modification effect, we computed the analytical resonant frequencies under various inner radii of the rib structure, employing the parameters of PZT and AlN^31^, two commonly utilized piezoelectric materials (refer to Table 1), as illustrated in Fig. 2a.

**Table 1:** Material properties of piezoelectric films^6^

Material	*ρ* (g/cm^3^)	*E* (GPa)	*d*_31_ (pC/N)	*ε*_33,*r*_
Poly-PZT	7.7	96	−130	1300
Single Crytal -PZT	—	—	−110	200
AlN	3.26	330	−2.65	9.5
Sc_9.5%_AlN	3.52	250	—	10.5

The PZT can provide a wider frequency modification range than AlN. When the rib structure is unetched (R = 0), the center frequencies for PZT and AlN are 85 kHz and 113 kHz, respectively. An increase in the inner radius of the rib structure causes partial etching of the piezoelectric layer and a decrease in film quality; additionally, the stiffness retention effect of the rib structure becomes apparent. Consequently, although the film mass is significantly reduced, the stiffness remains relatively unchanged, leading to increases in the resonant frequency and bandwidth. The peak values are observed at 300 kHz and 230 kHz for PZT and AlN, respectively. However, as the radius of the rib structure continues to increase, the stiffness attenuation becomes more pronounced, causing a decrease in the resonant frequency and bandwidth. This phenomenon is related to the stiffness and mass changes of the film, and the resonant frequency of the film is positively related to the stiffness and negatively related to the mass. When the inner radius first increases, the mass continues to decrease, and the stiffness is basically unchanged; thus, the resonance frequency increases. When the inner radius continues to increase, although the mass still decreases, the film stiffness rapidly decreases; thus, the resonance frequency decreases. The range of frequency regulation (Δf) and its percentage relative to the unetched resonant frequency (Δf/f_0_*100%) are determined to be {18.88 kHz, 24.9%} for PZT and {6.47 kHz, 5.8%} for AlN. Since PZT demonstrates a better frequency modification capability, it was selected as the preferred piezoelectric material used in this study.

### Analytical model of output charge

To further calculate the theoretical output charge of rib-structured microphones, an analytical model was established. Considering a uniform external sound pressure, Eq. (9) can be derived as follows:

12 $$\int D{\left({\nabla }^{2}{w}_{0}\right)}^{2}+T{\left(\nabla {w}_{0}\right)}^{2}{dS}+p{w}_{0}{dS}=\int \sigma {\omega }^{2}{w}_{0}^{2}{dS}$$

**Fig. 3:**
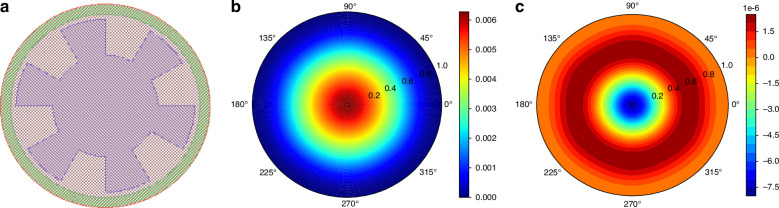
The validity test of the analytical model. **a** Rib-structure microphone layout with a 200$\,{\rm{\mu }}{\rm{m}}$ inner radius. **b** Vibration shape of diaphragm. **c** Validity test of model; a higher frequency input pressure field is tested and stimulates the “31” mode of the membrane

The membrane design and the vibration shape under input sound pressure are shown in Fig. 3a, b. The deformation at the edge of the diaphragm where the electrode is located is almost rotationally symmetric. Therefore, only the curvature in the radius direction is used to calculate the deformation of the piezoelectric material, and ultimately, the output charge can be calculated as follows:

13 $$Q=\frac{E}{1-\nu }\cdot \frac{{d}^{2}}{d{r}^{2}}\left(w\left(r\right)\right)\cdot \left({z}_{{piezo}}-{z}_{{central\; axis}}\right)\cdot {d}_{31}\cdot \pi \left({R}_{{out}}^{2}-{R}_{{in}}^{2}\right)$$

where $E$ and $\nu$ are the Young’s modulus and Poisson’s ratio of the piezoelectric material, respectively; $w(r)$ is the displacement resulting in the radius direction; ${d}_{31}$ is the piezoelectric factor of the material; ${z}_{{piezo}}$ is the height of the center of the piezoelectric layer; ${z}_{{central\; axis}}$ is the height of the stress neutral plane; and $S=\pi ({R}_{{out}}^{2}-{R}_{{in}}^{2})$ is the area of the electrode.

Moreover, a validation experiment for the numerical method was conducted. A higher frequency (approximately 250 kHz) pressure signal was used as the test input, and the calculation result of the vibration shape is shown in Fig. 3c; this result is consistent with the expected higher-order mode. After Eq. (13) was used to convert the vibration shape to the output charge of the electrode, the electrical output was finally obtained from the basic physical principles of diaphragm vibration and piezoelectricity. The final calculation results of our model are provided in Table 2, and they are in agreement with the FEM simulation results, which are approximately 30$\,\mu V$.

**Table 2:** Calculation results of the output charge

Number	Inner radius (*μm*)	Output Voltage (*μV*)
1	50	32.371
2	100	32.420
3	150	32.519
4	200	32.692
5	250	32.895
6	300	32.795

### Finite element model simulation

A finite element model was established based on the designed rib structure microphone, and a frequency domain simulation was carried out to verify the frequency regulation behavior of the rib structure microphone. As shown in Fig. 2b, by changing the inner radius R of the rib structure microphone, the resonant frequency of the microphone was changed from 72.8 kHz to 107.5 kHz; this result was basically consistent with the analytical model. In addition, the electrode width and thickness of the piezoelectric layer were optimized to obtain the best microphone performance. The electrode width was determined to be 10% of the cavity radius, and the PZT layer thickness was determined to be 1.1 μm with respect to a 2.2 μm Si matching layer.

### Fabrication process

The proposed microphone was fabricated using a standard MEMS process. The process flow is shown in Fig. 2c, which is divided into six steps: (i) The process started with an SOI wafer, with a top silicon layer thickness of 2.2 μm as the matching layer and a buried oxide layer thickness of 1 μm as the self-stopping layer of deep reactive ion etching (DRIE). (ii) 0.1 μm SiO_2_ was deposited on the surface as the electrical insulation layer, with 0.1 μm Pt as the bottom electrode and 0.1 μm LNO (LaNiO_3_) as the lattice matching layer for high-quality PZT deposition; (iii) 1.1 μm single-crystal PZT was deposited as a piezoelectric layer with a d_31_ greater than 110 pC/N and a dielectric coefficient of no more than 200 pC/N, which indicated that it was well suited for sensing mode operation; (iv) PZT was patterned by wet etching (HF+HCl mixed solution) to form the rib-structures; (v) After etching was completed, 50 nm Ti and 150 nm Al were deposited and lifted off on the surface as wires and electrodes; (vi) With the buried oxide layer as a stop layer, the cavity was etched by DRIE on the back of the wafer.

### Experiment

#### XRD test

**Fig. 4:**
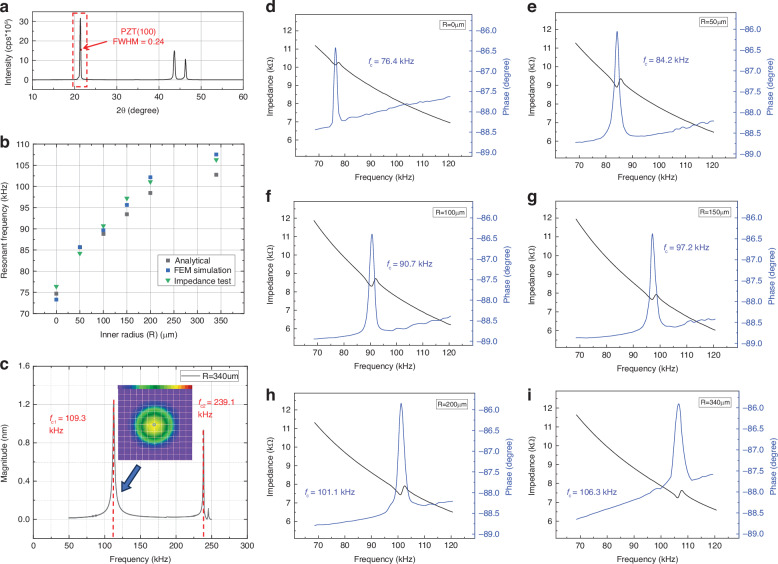
Impedance and LDV test results. **a** An X-ray Diffraction (XRD) results of the single crystal PZT film. **b** Analytical model, FEM, and the measurement results of the resonant frequency change with the inner radius change. **c** LDV test results of the microphone with an inner radius of 340 μm. **d**–**i** Electric impedance measurement results of the different rib-structure microphones with various inner radius, and the resonant frequency varies from 76.4 kHz to 106.3 kHz while the inner radius is changed from 0 μm to 340 μm

The single-crystal PZT layer was measured via XRD, as shown in Fig. 4a. The scanning range is 10–60°, covering all possible ranges of the PZT crystal orientation. A significant PZT (100) diffraction peak is observed at 21°, and no other crystal orientations of PZT are observed at other angles. The FWHM is measured via Gaussian function fitting using MATLAB, and the result is 0.24°, which verifies the good (100) crystal orientation of the PZT.

#### Impedance Test

To measure the resonant frequencies of the six fabricated rib-structure microphones and verify the feasibility of the rib-structure for frequency modification, an impedance analysis AGILENT 4294 A was used to measure the impedance and phase angle curves of the microphones.

The sweep frequency range was set to 60–130 kHz according to the simulation results, and the sweep frequency adopted a small AC signal with an amplitude of 0.5 mV. The black curve represents the impedance curve, and its amplitude decreases with increasing frequency, indicating capacitance characteristics. At the resonant frequency, the system energy dissipation is minimal, and the device impedance is minimal; thus, this corresponds to the valley value point^32^. The blue curve represents the angle between the current component and the voltage component of the device impedance, that is, the phase angle. When the frequency is low, the microphone is similar to the capacitor, and the voltage component lags behind the current component by 90°; here, the phase angle is −90°. As the frequency increases, the capacitance decreases, the voltage component moves closer to the current component, and the phase angle gradually decreases and shifts from −90° to 0°^33^. The impedance axis is gradually approached by the second quadrant in the impedance admittance plane. When resonance occurs, a phase angle jump occurs^34^, and then the trend continues to decrease. Figure 4d–i shows the six types of microphones with different rib lengths. As the rib length decreases, the quality of the film decreases; hence, the resonant frequency increases, corresponding to an increase in the valley point of the impedance curve in the figure. The frequency range regulated by the rib structure design is from 76.4 kHz to 106.3 kHz. The sensitivity can potentially be increased by reducing the modulus at the same resonant frequency. The LDV test results and the vibration mode shapes are shown in Fig. 4c and verify that the peak values in Fig. 4c–h correspond to the fundamental resonant mode. A minor frequency difference is observed between the impedance and LDV measurement results, which may be due to the difference in the test setup routing parasitic and resistance and mechanical and acoustic damping effects.

The measured results of the characteristic frequency are compared with those of the analytical model and the finite element simulation model. As shown in Fig. 4b, as the inner radius of the rib–structure microphones increased, the residual mass decreased from 100% to 60%, and the resonant frequencies of the analytical model, the finite element model, and the measured results consistently increased. The three models were basically consistent, but few differences existed. The FEM simulation results showed a larger frequency modification range than the analytical results because nonuniform internal stress was present between the different layers.

#### Capacitance extraction

The impedance results were also used to calculate the output capacitance of the microphone according to the circuit model and could provide a reference for amplifier design. The circuit model of the proposed rib structure microphone is shown below, where R represents the leakage resistance and C represents the capacitance of piezoelectric devices. The desired information is showed by the charge accumulated on the capacitor. Due to the relationship between charge and current, replacing the charge source with a current source can greatly facilitate further simulations.

The specific values of both R and C can be estimated by measuring the total impendence of the device using an impendence analyzer and determining the best-fit parameters with an algorithm. Typically, the leakage resistance ‘R’ is sufficiently large and can be disregarded in designs that do not require high accuracy, whereas the capacitance ‘C’ is always critical in most designs due to its significant role in resonance^35–37^.

**Fig. 5:**
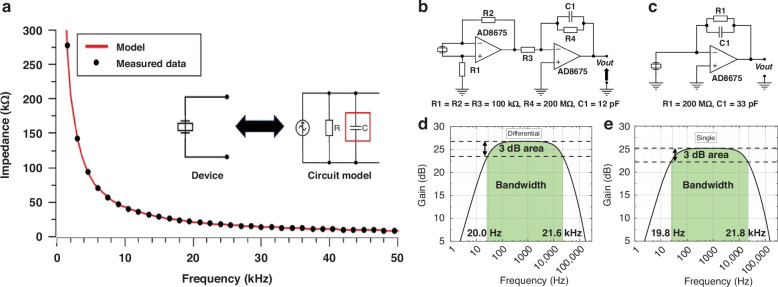
Schematic of the amplifiers design. **a** Typical model of the piezoelectric microphone and an example of the capacitance extraction model. **b**, **c** Two-stage differential charge amplifier and the single-ended charge amplifier. **d**, **e** Frequency response simulation results from the differential and single-ended charge amplifiers

The impedance measurements were obtained using the AGILENT 4294 A impedance analyzer; the analyzer is used to measure the variation of the impedance in the frequency range from 40 Hz to 300 kHz. By employing the least-squares method, we extracted the optimal parameters from the collected data. Figure 5a shows an instance of this modeling, where the model aligns perfectly with the data. The relative error of each sample does not exceed 1% within 50 kHz and 2% within 300 kHz. Additionally, the estimated capacitance is also in good agreement with the calculated value based on the dielectric constant 200, which verifies the accuracy of the circuit model and simulation.

#### Design of the charge amplifier

Charge amplifiers convert electrical charge signals into voltage signals, which are integral to piezoelectric designs. They are typically positioned at the initial stage of data acquisition systems, highlighting the importance of designing high-performance charge amplifiers. The primary principle of a charge amplifier involves the use of negative feedback from a capacitor to transform charge signals into voltage signals^38^. The gain is determined by the ratio of the sensor’s capacitance to the feedback capacitance.

Charge amplifiers can be categorized on the basis of their input signals into single-ended and differential types; notably, differential amplifiers provide significant advantages over single-ended amplifiers, particularly in terms of noise considerations. In a single-ended amplifier, both the interfering noise and the signal are amplified; however, in the differential outputs, the common mode interfering noise applied to the two ports cancel each other, resulting in a higher signal-to-noise ratio^39,40^. In addition to noise considerations, differential charge amplifiers can provide other advantages, such as reduced distortion, balanced outputs, and increased gain. Here, noise is specifically highlighted because it is relevant to the microphone SNR.

Conventional differential charge amplifiers typically have a limited CMMR, which is highly dependent on the matching of resistors and capacitors^41^. In this study, a two-stage differential charge amplifier was specifically designed to obtain better performance, as shown in Fig. 5b. The common-mode input is rejected by the first stage, and the integration is independently carried out by the second stage. A single-ended charge amplifier was also designed, as shown in Fig. 5c, for comparison. The simulation results of the frequency response using LTspice are shown in Fig. 5d, e and verify that the proposed circuit design can support a bandwidth of 20 Hz to 20 kHz. Both the differential and the single-ended amplifiers can cover the 20 Hz–20 kHz bandwidth within a 3 dB gain reduction. Although they have similar bandwidths, the differential amplifier is a better choice because it can eliminate common-mode noise and provide a higher gain.

#### Acoustic Test

**Fig. 6:**
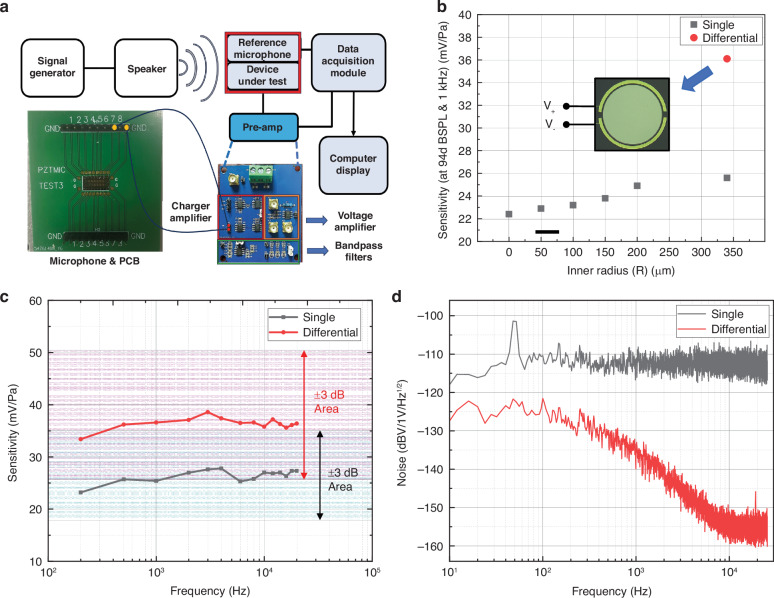
Acoustic test results of the microphone. **a** Designed acoustic test system for rib-structure microphones and differential output microphone. **b** Sensitivities of microphones with different rib structures and differential outputs at 94 dB SPL and 1 kHz. **c** Sensitivity variation of single-ended and differential microphones in the audio bands. **d** Comparison of the A-weighted noise amplitude spectral density for single and differential microphones

To evaluate the acoustic characteristics of the prepared microphone and confirm the effectiveness of the rib structures for frequency adjustment, we utilize the test system illustrated in Fig. 6a. The entire system includes a signal generator, a speaker, rib-structure microphones, a reference microphone (B&K 4138), the designed charge amplifier, a data acquisition module, and a display terminal. Moreover, a voltage amplifier is added to boost the signal, a bandpass filter is used to filter out noise, and the entire test system is placed in a Faraday cage to isolate electromagnetic noise.

First, the sensitivity at 1 kHz and 94 dB SPL of the six designed rib-structure microphones with different inner radii was measured; these data provide a reference for the change in stiffness. As shown in Fig. 6b, the sensitivity of the six rib-structure microphones minimally changed, from only 22.3 mV/Pa for R = 0 μm to 25.7 mV/Pa for R = 340 μm; these results confirmed the retention of stiffness by the rib structures. A differential output microphone with R = 340 μm was also tested. The annular sensing electrode was divided into two identical parts. A voltage of 20 V was applied to pole one part, whereas −20 V was used to attain reverse polarization for the other part. The two electrodes then formed a differential output together with the bottom electrode. According to the test results, the differential device could achieved a sensitivity of 36.1 mV/Pa; this value was a significant improvement over the single-ended sensitivity of 25.7 mV/Pa.

To further illustrate the performance of the microphones in the audio bandwidth, we tested the single-ended microphone and the differential output microphone by sweeping the frequency from 200 Hz to 20 kHz at 94 dB SPL; both have an R of 340 μm, as shown in Fig. 6c. The change in output sensitivity always remained less than ±3 dB for both devices, and the differential output microphone showed a higher sensitivity than the single-ended microphone. Due to the bandwidth of the speaker, we did not test the microphones at higher frequencies; however, the results of the impedance test indicated that the bandwidth of the microphone could be greater, especially since the rib structures could cause an increase in the resonant frequency. The A-weighted noise amplitude spectral density for single and differential microphones was also compared. The noise floor over the audio band was determined to be −82.8 dBV for the differential devices and −68.2 dBV for the single-ended devices, as shown in Fig. 6d. Due to reduced common mode noise, microphones with a differential output had lower noise than the microphones with a single-ended output, particularly over 100 Hz. However, when the frequency was lower than 100 Hz, the 50 Hz electromagnetic background noise and its harmonics became dominant. Although we made a Faraday cage to reduce the background noise, electromagnetic interference could enter the circuit from the input ports of the preamplifier, as shown in Fig. 6d. When the frequency exceeded 100 Hz, the interference caused by the electromagnetic signal rapidly decreased; thus, the noise difference between the differential and the single-ended outputs became evident. As a result, the SNR increased from 36.4 dB to 53.9 dB with the differential output, and the performance of the designed microphone was comparable to those in other studies, as shown in Table 3.

**Table 3:** Comparison of the various kinds of microphones

Literature	Material	Sensitivity	SNR	Bandwidth	Output
Reference^6^	Poly-PZT	1.6 μV/Pa	N/A§	100 Hz-50 kHz	Single-ended
Reference^6^	AlN	39 μV/Pa	N/A§	69 Hz-20 kHz (*f*_c_=104 kHz)	Single-ended
Reference^25^	Sc_9.5%_AlN	0.206 mV/Pa	54.2 dB	N/A§	Single-ended
This work	Single-crystal PZT	25.7 mV/Pa*	36.4 dB	200 Hz-20 kHz (*f*_c_=106 kHz)	Single-ended
This work	Single-crystal PZT	36.1 mV/Pa*	53.9 dB	200 Hz-20 kHz (*f*_c_=106 kHz)	Differential

§ Not available * With amplifier *f*_*c*_ Device resonant frequency

## Conclusion

In this study, the design, fabrication, and testing of a piezoelectric microphone with a high-quality single-crystal PZT film is presented. By constructing rib structures to maintain stiffness, the resonant frequency can be increased by reducing the mass, and the bandwidth can be expanded. A resonant frequency tuning range of 76.4 kHz-106.3 kHz was verified by the impedance tests, and a corresponding analytical model was created and verified using FEM simulations and the experimental results. Due to the good agreement, the computing time of the analytical method was greatly reduced compared with that of the FEM. The microphone with a 340 μm inner radius shows the optimal sensitivity and the highest resonant frequency of 106.3 kHz, which indicates a potentially greater linearity under high SPL. Additionally, to improve the SNR, a strategy of differential outputs was developed based on the polarization characteristics of the PZT, and a two-stage differential charge amplifier was designed. Compared with the single-ended microphone, the differential microphone can increase the sensitivity from 25.7 mV/Pa to 36.1 mV/Pa, whereas the background noise is reduced from −68.2 dB to −82.8 dB; this results in an increase of the SNR from 36.4 dB to 53.9 dB. Although the resonant frequency of the microphone in this study exceeds that of the audio band, our results can provide a reference for the design of the microphone in application scenarios with high SPL and other requirements for stiffness; moreover, reducing the modulus and improving the sensitivity for the same resonant frequency is possible and is usually indicative of the AOP. In addition, the method and analytical model of maintaining stiffness to bandwidth modification presented in this study can also optimize the design of the bandwidth product of the SPL. Furthermore, this strategy may provide guidance for the design of directional microphones, particularly in expanding the bandwidth. In this study, the feasibility of piezoelectric microphones based on single-crystal PZT is demonstrated, a method to adjust the frequency and bandwidth using rib structures is proposed, and advanced analytical modeling and solution methods are provided, with valuable insights for the design of piezoelectric microphones.
